# The Theory and Practice of Infant Feeding

**Published:** 1902-10

**Authors:** 


### BOOK REVIEWS.

THE THEORY AND PRACTICE OF INFANT FEEDING. With Notes on Development. By HENRY DWIGHT CHAPIN, A. M., M. D., Professor of Diseases of Children at the New York Post-Graduate Medical School and Hospital, etc. Octavo, pp. 335. Illustrated. New York: William Wood & Company. 1902. [Price, $2.25 net.]

The plan which Dr. Chapin has pursued in this work on infant feeding has been not simply to give various formulæ with indications for their use, but to set forth the principles which underlie the successful feeding of infants leaving the physician for the most part to apply these for himself. · The book is divided into four parts. In the first, the digestive systems, foods, and processes of digestion in the common domestic animals—dogs, horse and cow—are compared, and the adaptation of the digestive tract of each to the food of the animal is shown. Analyses of the milk of each species are given together with its manner of coagulation, and attention is called to the resemblance which exists between the food of the adult and the young animal of each class and the bearing this has upon the development of the digestive class.

Part II. takes up the subject of raw food materials. The first chapter is occupied with analyses of cow's milk, the variations to which it is subject, the nature of the proteids, cream, gravity and centrifugal, and analyses of condensed milk. Then follows a chapter on the bacteriology of milk which is supplemented by a concise and practical chapter on the bacteriological examination of milk by Prof. Conn, of Wesleyan University. This, as well as the chapter on the quantitative analysis of milk is complete enough in the methods given to be of value and yet not so complex as to be beyond the skill of an ordinarily intelligent person. The preservation of milk, the character of market milk and certified milk complete the topics here considered.

Part III. is devoted to infant feeding proper. Breast feeding is briefly considered. The burden of Dr. Chapin's argument in regard to infant feeding is in substance this: "It must be clearly borne in mind that the nutrition and development of the digestive tract must be considered together. Little progress will be made if only a calculation of the composition of food is made."

This section is an admirable one. Many methods for modifying milk with anything like accuracy are so complex as to be disheartening. The method here presented is simplicity itself. As diluents, Dr. Chapin prefers the cereal waters, both because of their effect on the curd and because of the nourishment they add to the mixture.

The concluding section of the book is devoted to the growth and development of the normal infant which by comparison affords the best test of successful artificial feeding. For this part of the work 200 carefully selected healthy infants, ranging in age from a few hours to two years, were examined by the anthropologist, Dr. Hedlicka. The tables prepared from his measurements should be of much value to the pediatrist.

This work places the subject of infant feeding on a broader, more scientific basis than any of its predecessors have done. If some of its statements cannot yet be regarded as final, they are rational and the book, as a whole, is eminently satisfactory.

The mechanical part is worthy of mention for its excellence.

